# Digital Twin-Enabled Extrusion Control for High-Fidelity Printing of Polymers

**DOI:** 10.3390/polym17162215

**Published:** 2025-08-13

**Authors:** Kantawatchr Chaiprabha, Chaiwuth Sithiwichankit, Worathris Chungsangsatiporn, Gridsada Phanomchoeng, Ratchatin Chancharoen

**Affiliations:** 1 Department of Mechanical Engineering, Faculty of Engineering, Chulalongkorn University, Bangkok 10330, Thailandchaiwuth.s@chula.ac.th (C.S.);; 2 Micro/Nano Electromechanical Integrated Device Research Unit, Faculty of Engineering, Chulalongkorn University, 254 Phaya Thai Road, Bangkok 10330, Thailand

**Keywords:** polymer direct ink writing, rheology-variant material, digital twin, dual-mode control for additive manufacturing

## Abstract

Direct ink writing (DIW) has emerged as a powerful technique for functional-structure fabrication. However, its application to materials with heterogeneous or time-dependent rheology remains limited. This study introduces dual-mode electropneumatic extrusion, supported by a real-time digital twin. This platform integrates a motorized pneumatic cylinder with an electropneumatic pressure regulator, enabling continuous blending of pressure and displacement control. System performance is evaluated across five material characteristics: homogeneity, heterogeneity, time-dependent rheology, self-curing ability, and thermoplasticity. The results demonstrate that feedback current control reduces the linewidth variability to ≈2% and settling time to <250 ms, even under four-fold increases in viscosity. Adaptive pressure ramps restore variability to ≤4% throughout material curing, while hybrid velocity–pressure operation maintains variability at ≤4% and a pore geometry error below 4% over 20 layers. These findings establish a robust framework for rheology-adaptive DIW and offer practical guidelines for implementing dual-mode control in high-throughput, multi-nozzle applications.

## 1. Introduction

Liquid deposition modelling, commonly referred to as direct ink writing (DIW), has emerged as a versatile extrusion-based additive manufacturing technique for liquid and paste-like materials [1,2,3]. DIW offers high customizability and rapid prototyping, enabling the fabrication of complex geometries that are difficult to achieve with traditional manufacturing methods. DIW’s versatility spans an extraordinary range of materials, such as soft polymers [4], biomaterials [5], ceramics [6], metals [7], composites [8,9], and even foodstuffs [10], supporting advances in bioengineering [11,12], flexible electronics [13], and beyond. Despite this flexibility, DIW remains limited by challenges in achieving consistent extrusion accuracy, especially when handling materials with evolving or nonuniform rheological behavior.

**Figure 1:**
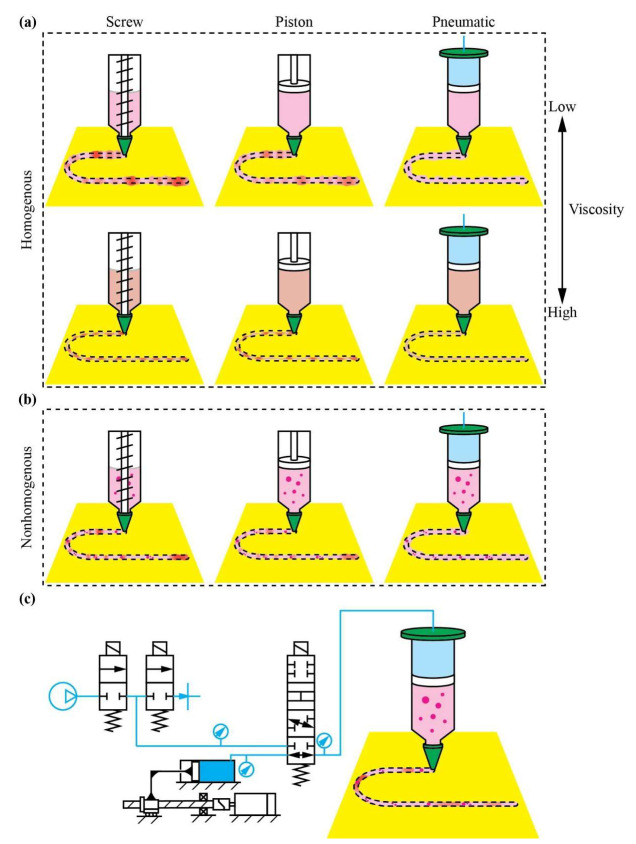
Landscape of material extrusion in DIW: (**a**) conventional methods on homogeneous materials, (**b**) conventional methods on nonhomogeneous materials, and (**c**) dual-mode electropneumatic extrusion on nonhomogeneous materials.

The steadiness of material flow, which critically influences strand fidelity, pore geometry, and overall print quality, is governed by the interaction between material rheology and extrusion mechanics [14,15]. Most DIW inks exhibit viscoelasticity, with the shear modulus and viscosity significantly varying under different conditions: namely, temperature, pressure, and shear rate [16]. Achieving stable and repeatable deposition requires precise regulation of both transient and steady-state responses, such as suppressing overshoot, minimizing settling time, and maintaining a uniform linewidth. Conventional extrusion methods, including pneumatic syringes [17], piston dispensers [18], and screw extruders [19], rely on either pressure [20] or feed rate [21] control (Figure 1). Pneumatic systems provide a lightweight printhead and inherent pressure visibility. However, they struggle to maintain volumetric consistency when ink properties shift. On the other hand, piston and screw drives deliver a reliable average flow but lack native pressure sensing and suffer from mechanical inertia and thermal constraints. Crucially, none of these approaches can reliably process materials that require both precise thermal management and real-time adaptation to rapidly evolving viscoelasticity, such as two-component resins, self-curing sealants, or thermoplastic polymers [22,23].

To bridge this gap, several approaches have been proposed. Iterative learning of the pressure–flow relationship has been applied to micro-extrusion systems to improve flow precision [21], while pressure advance algorithms have been used to compensate for pneumatic lag during directional changes [24]. Feedback-driven extrusion using current sensing or displacement tracking has also been introduced to improve response time and deposition consistency [22]. More recently, efforts have been made to incorporate model-based control frameworks that account for material properties during printing [15,23]. However, most of these systems are tailored to either pressure or feed rate regulation alone and lack the ability to seamlessly accommodate dynamic changes in material rheology. Moreover, the use of real-time digital twin models, capable of simulating the combined electrical, mechanical, pneumatic, and rheological domains, has not been fully leveraged for extrusion control.

This study introduces a digital twin-enabled dual-mode electro-pneumatic extrusion platform for rheology-adaptive DIW (Figure 1). A supervisory scheduler manages four control schemes: (1) pressure regulation using a proportional valve, (2) PID control of syringe velocity for volumetric flow, (3) an open-loop mode for direct pressure and flow setting, and (4) a combined mode that integrates both feedback loops. With such control schemes, dynamic rheological changes are accommodated. To accomplish online parameter identification and state estimation across extensive domains (electrical, mechanical, pneumatic, and rheological), a Simscape-based digital twin model was proposed, ensuring continuous synchronization between the virtual model and physical system. Five benchmark experiments were conducted to validate the proposed architecture, using materials with varying properties, including a homogeneous acrylic paint, a paint mixture with an uneven concentration, a curing silicone resin (RUNGART, Infinite Craft Co., Ltd., Bangkok, Thailand), a self-curing acrylic sealant, and thermoplastic polypropylene. Across all cases, the results demonstrate significant reductions in linewidth variability and settling time compared to conventional single-mode control strategies, even under rapidly changing material conditions. This work presents a unified framework for real-time, material-adaptive DIW extrusion and establishes new benchmarks for accuracy control through hybrid actuation and digital twin integration.

## 2. System Design and Control Architecture

### 2.1. Material Rheology

**Figure 2:**
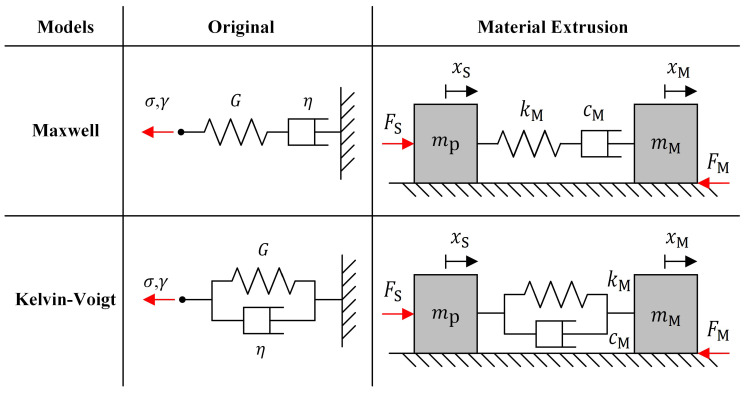
Mechanical analogies of the Maxwell and Kelvin–Voigt viscoelastic models.

Rheology is the field of study that examines material deformation and flow, particularly the relationship between shear stress σ and strain γ. In elastic materials, σ is proportional to γ, expressed as $\sigma \left(t\right)=G\left(t\right)\gamma \left(t\right)$, where G is the shear modulus. In contrast, viscous materials follow a different relationship, where σ is proportional to $\dot{\gamma }$. This is expressed as $\sigma \left(t\right)=\eta \left(t\right)\dot{\gamma }\left(t\right)$, where η represents the viscosity of the material. DIW printing materials are typically pastes and gels that exhibit both elastic and viscous behavior and are thus known as viscoelastic. Traditional viscoelastic models include the Maxwell and Kelvin–Voigt models. The Maxwell model characterizes σ relaxation under constant γ, while the Kevin–Voigt model describes γ creep under constant σ (Figure 2) [25]. Viscoelastic behavior can be represented using springs and dampers. In the Maxwell model they are arranged in series. In the Kevin–Voigt model, they are arranged in parallel. Notably both G and η vary under different conditions, such as temperature and pressure.

This study employs the Maxwell and Kelvin–Voigt models to characterize material behavior during extrusion. In Figure 2, the syringe piston and printing material are modeled as masses $m_{P}$ and $m_{M}$ with displacements $x_{S}$ and $x_{M}$, respectively. Material elasticity is represented by a spring stiffness $k_{M}$, while η is portrayed by a damper coefficient $c_{M}$. The piston applies a driving force $F_{S}$ that is countered by the material’s resistance $F_{M}$ due to its elastic and viscous properties. Herein, $F_{M}$ is modelled as $F_{M}\left(t\right)={f_{T}\left(t\right)+c}_{O}\left(t\right){\dot{x}}_{M}\left(t\right)$, where all coefficients depend on material conditions. Let $F_{V}$ denote an internal force arising from the material’s viscoelasticity. The Maxwell-based formulation can be written as

(1) $$\left[\begin{array}{c} {\ddot{x}}_{S}\left(t\right)\\ {\ddot{x}}_{M}\left(t\right)\\ {\dot{F}}_{V}\left(t\right) \end{array}\right]=\left[\begin{array}{c} \frac{{-F_{V}\left(t\right)+F}_{S}\left(t\right)}{m_{P}}\\ \frac{{-f_{T}\left(t\right)-c_{O}\left(t\right){\dot{x}}_{M}\left(t\right)+F}_{V}\left(t\right)}{m_{M}\left(t\right)}\\ k_{M}\left(t\right)\left({\dot{x}}_{S}\left(t\right)-{\dot{x}}_{M}\left(t\right)\right)-\frac{k_{M}\left(t\right)F_{V}\left(t\right)}{c_{M}\left(t\right)} \end{array}\right],$$

The Kelvin–Voigt model, which integrates elastic and viscous effects in parallel, can be written as

(2) $$\left[\begin{array}{c} {\ddot{x}}_{S}\left(t\right)\\ {\ddot{x}}_{M}\left(t\right) \end{array}\right]=\left[\begin{array}{c} \frac{{-k}_{M}\left(t\right)\left(x_{S}\left(t\right)-x_{M}\left(t\right)\right)-c_{M}\left(t\right)\left({\dot{x}}_{S}\left(t\right)-{\dot{x}}_{M}\left(t\right)\right)+F_{S}\left(t\right)}{m_{P}}\\ \frac{{-f_{T}\left(t\right)-c_{O}\left(t\right){\dot{x}}_{M}\left(t\right)+k}_{M}\left(t\right)\left(x_{S}\left(t\right)-x_{M}\left(t\right)\right)+c_{M}\left(t\right)\left({\dot{x}}_{S}\left(t\right)-{\dot{x}}_{M}\left(t\right)\right)}{m_{M}\left(t\right)} \end{array}\right].$$

Equations (1) and (2) indicate that the material velocity ${\dot{x}}_{M}$ lags behind the piston velocity ${\dot{x}}_{S}$. The relationship between ${\ddot{x}}_{S}$ and ${\ddot{x}}_{S}$ can also be formulated as

(3) $${\ddot{x}}_{M}\left(t\right)=\frac{-f_{T}\left(t\right)-c_{O}\left(t\right){\dot{x}}_{M}\left(t\right){-m}_{P}{\ddot{x}}_{S}\left(t\right)+F_{S}\left(t\right)}{m_{M}\left(t\right)}.$$

At steady state, $F_{S}\left(t\right)=f_{T}\left(t\right)+c_{O}\left(t\right){\dot{x}}_{M}\left(t\right)$. The convergence of ${\dot{x}}_{S}$ and ${\dot{x}}_{M}$ is implied. Under pressure control, input changes follow step variations in $F_{S}$. In feed rate control, they follow corresponding changes in ${\dot{x}}_{S}$.

### 2.2. Pneumatic Transmission

**Figure 3:**
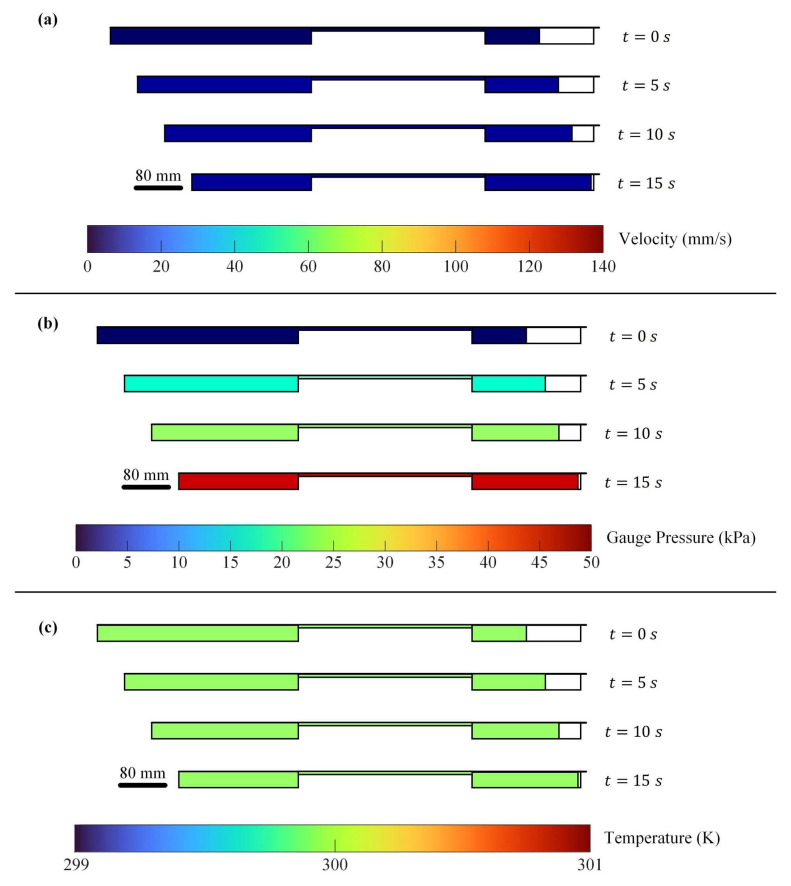
Simulation results illustrating the profiles of air velocity, gauge pressure, and temperature during the extrusion of a custom liquid material, used to determine transient gradients of air properties in pneumatic transmission: (**a**) speed profile, (**b**) Gauge pressure profile, and (**c**) temperature profile.

Pneumatic transmission transfers power using compressible air, providing mechanical flexibility between the driving and driven components. In this study, a pneumatic cylinder drives a syringe, with a dead-volume chamber linking the two components. According to the Navier–Stokes equations [26], air properties (velocity, pressure, and density) are governed by simultaneous nonlinear dynamics [27]. To analyze transient air gradients, a 2D axisymmetric simulation of liquid extrusion was conducted, incorporating laminar flow and heat transfer. Table 1 lists the simulation parameters. In Figure 3, the simulation results indicate that pressure and temperature gradients are negligible when velocity differences are below 140 mm/s, and temperature remains constant.

Under uniform air conditions, the mass of air in the cylinder $m_{C}$, syringe $m_{S}$, and dead volume $m_{D}$ depends on their respective pressures ($P_{C}$, $P_{S}$, and $P_{D}$), temperatures ($T_{C}$, $T_{S}$, and $T_{D}$), and volumes ($V_{C}$, $V_{S}$, and $V_{D}$). Mass flow rates can be expressed as

(4) $$\left[\begin{array}{c} {\dot{m}}_{C}\left(t\right)\\ {\dot{m}}_{D}\left(t\right)\\ {\dot{m}}_{S}\left(t\right) \end{array}\right]=\left[\begin{array}{c} \frac{\partial m_{C}}{\partial P_{C}}{\dot{P}}_{C}\left(t\right)+\frac{\partial m_{C}}{\partial T_{C}}{\dot{T}}_{C}\left(t\right)+\frac{\partial m_{C}}{\partial V_{C}}{\dot{V}}_{C}\left(t\right)\\ \frac{\partial m_{D}}{\partial P_{D}}{\dot{P}}_{D}\left(t\right)+\frac{\partial m_{D}}{\partial T_{D}}{\dot{T}}_{D}\left(t\right)+\frac{\partial m_{D}}{\partial V_{D}}{\dot{V}}_{D}\left(t\right)\\ \frac{\partial m_{S}}{\partial P_{S}}{\dot{P}}_{S}\left(t\right)+\frac{\partial m_{S}}{\partial T_{S}}{\dot{T}}_{S}\left(t\right)+\frac{\partial m_{S}}{\partial V_{S}}{\dot{V}}_{S}\left(t\right) \end{array}\right].$$

With the enclosed air, the total mass flow satisfies ${\dot{m}}_{C}\left(t\right)+{\dot{m}}_{D}\left(t\right)+{\dot{m}}_{S}\left(t\right)=0$. Additional constraints are $P_{C}\left(t\right)=P_{D}\left(t\right)=P_{S}\left(t\right)=F_{C}\left(t\right)/A_{C}=F_{S}\left(t\right)/A_{S}$ and ${\dot{T}}_{C}\left(t\right)={\dot{T}}_{D}\left(t\right)={\dot{T}}_{S}\left(t\right)=0$. Using these constraints, piston and syringe velocities can be determined as

(5) $${\dot{x}}_{S}\left(t\right)=\frac{A_{C}{\dot{x}}_{C}\left(t\right)}{A_{S}}-\frac{P_{S}\left(0\right)\left(A_{C}l_{C}+V_{D}+A_{S}l_{S}\right){\dot{F}}_{S}\left(t\right)}{A_{S}A_{C}{\left(P_{atm}+\frac{F_{C}\left(t\right)}{A_{C}}\right)}^{2}},$$

where $P_{atm}$ is atmospheric pressure and $l_{C}$, $l_{S}$, $D_{C}$, and $D_{S}$ denote the cylinder and syringe dimensions. Force transmission can be derived as

(6) $$F_{S}\left(t\right)=\frac{D_{S}^{2}F_{C}\left(t\right)}{D_{C}^{2}}.$$

Equation (5) indicates a time delay in ${\dot{x}}_{S}$ relative to ${\dot{x}}_{C}$. Since $P_{S}$ must remain positive, pneumatic transmission is inherently asymmetric and delayed. While $P_{S},\;F_{S}$, and ${\dot{F}}_{S}$ can be measured, ${\dot{x}}_{S}$ is difficult to capture due to syringe space constraints. Therefore, the use of air models are recommended to estimate $l_{S}$, thereby allowing for the indirect evaluation of $x_{S}$.

### 2.3. Hardware Architecture

**Figure 4:**
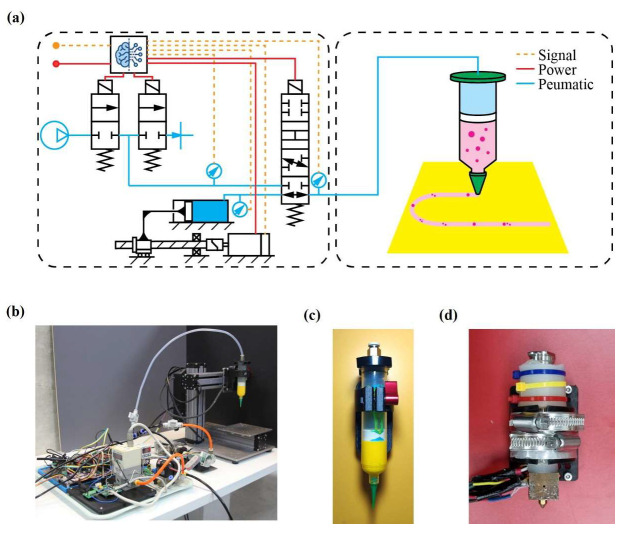
Dual-mode electropneumatic extrusion architecture: (**a**) schematic diagram, (**b**) photograph, (**c**) room-temperature printhead with 30 mL capacity, and (**d**) high-temperature printhead with 10 mL capacity.

This section outlines the hardware architecture of an extrusion system with pneumatic transmission, enabling real-time control of $P_{S}$ and ${\dot{x}}_{M}$ (Figure 4a,b). The system employs a servo-driven ball screw to manipulate $P_{S}$, creating a pneumatic buffer that transmits thrust to the printhead. Unlike direct mechanical coupling, this configuration provides intrinsic overload protection, compliance, and $P_{S}$ monitoring capabilities. A supervisory scheduler dynamically blends two control schemes: (1) regulation of $P_{S}$, in which ${\dot{x}}_{M}$ is mainly inferred from $P_{S}$ values and nozzle geometries, and (2) control of $x_{C}$, in which piston travel enforces volumetric delivery to accommodate rapid changes in material rheology. Furthermore, a pressure advance algorithm [24] is exploited on an embedded microcontroller to compensate for the lags in $P_{S}$. The architecture separates the stationary components (motor, valves, and sensors) from the mobile printhead, reducing moving mass while maintaining full access to $P_{S}$ and $x_{S}$ data. This modular design supports various printhead configurations and operating conditions without requiring changes to the stationary components. Concurrent logging of key variables enables real-time diagnostics and digital twin calibration.

### 2.4. System Modeling and Stability Analysis

The complete system model was derived by integrating component-level models. By substituting displacement and force terms into Equation (3), ${\ddot{x}}_{M}$ can be expressed as a function of ${\dot{x}}_{M}$, motor torque $\tau_{R}$, and ${\ddot{\tau }}_{R}$:

(7) $${\ddot{x}}_{M}\left(t\right)=-\frac{f_{T}\left(t\right)+c_{O}\left(t\right){\dot{x}}_{M}\left(t\right)}{m_{M}\left(0\right)-\frac{D_{S}^{2}\rho_{M}\left(t\right)x_{M}\left(t\right)}{4}}+\frac{2a_{2}D_{S}^{2}\tau_{R}\left(t\right)}{a_{1}\left(m_{M}\left(0\right)-\frac{D_{S}^{2}\rho_{M}\left(t\right)x_{M}\left(t\right)}{4}\right)}\;\;+\frac{m_{P}D_{C}^{2}D_{W}\tan\left(\alpha \right)\tau_{R}\left(t\right)}{2J_{R}D_{S}^{2}\left(m_{M}\left(0\right)-\frac{D_{S}^{2}\rho_{M}\left(t\right)x_{M}\left(t\right)}{4}\right)}\;\;+\frac{32P_{S}\left(0\right)a_{2}\left(V_{D}+\frac{l_{C}D_{C}^{2}}{4}+\frac{l_{S}D_{S}^{2}}{4}\right){\ddot{\tau }}_{R}\left(t\right)}{{a_{1}D_{S}^{2}\left(P_{atm}+\frac{8a_{2}}{a_{1}}\right)}^{2}\left(m_{M}\left(0\right)-\frac{D_{S}^{2}\rho_{M}\left(t\right)x_{M}\left(t\right)}{4}\right)\tau_{R}\left(t\right)}.$$

Here, $a_{1}=D_{C}^{2}D_{W}\tan\left(\alpha +\beta \right)$, $a_{2}=1-a_{3}/J_{R}$, and $a_{3}=m_{W}\tan\left(\alpha +\beta \right)\tan\left(\alpha \right)D_{W}^{2}/4+J_{W}$. The variables $J_{R}$ and $J_{W}$ represent the moments of inertia of the motor and ball screw. The screw’s outer diameter is denoted by $D_{W}$. Moreover, α and β are the screw lead and friction angles, respectively. This nonlinear, second-order model incorporates multiple inputs and time-varying parameters.

To facilitate analysis, the model was linearized under the assumptions ${\ddot{\tau }}_{R}\left(t\right)=0$, $m_{M}\left(t\right)=m_{M}\left(0\right)$, $f_{T}\left(t\right)=f_{T}$, and $c_{O}\left(t\right)=c_{O}$. The resulting linear model can be written as

(8) $${\ddot{x}}_{M}\left(t\right)=-\frac{c_{O}{\dot{x}}_{M}\left(t\right)+f_{T}}{m_{M}\left(0\right)}+\frac{m_{P}{D_{W}D}_{C}^{2}\tau_{R}\left(t\right)}{2m_{M}\left(0\right)J_{R}D_{S}^{2}}\;\;+\frac{2D_{S}^{2}\left(1-\frac{m_{W}{\tan\left(\alpha \right)\tan\left(\alpha +\beta \right)D}_{W}^{2}+{4J}_{W}}{4J_{R}}\right)\tau_{R}\left(t\right)}{m_{M}\left(0\right)\tan\left(\alpha +\beta \right){D_{W}D}_{C}^{2}}.$$

The model has poles at 0 and $-c_{O}/m_{M}\left(0\right)$, indicating marginal stability for $x_{M}$ and asymptotic stability for ${\dot{x}}_{M}$. The model also confirms the system’s controllability with $\tau_{R}$ as input. However, due to practical variations in $m_{M}$, $f_{T}$, and $c_{O}$, the real system behaves more complexly than predicted. Consequently, achieving ${\dot{x}}_{M}$ stability remains a complex challenge, as it lacks a single definitive optimal solution.

### 2.5. Digital Twin Framework and Co-Simulation

**Figure 5:**
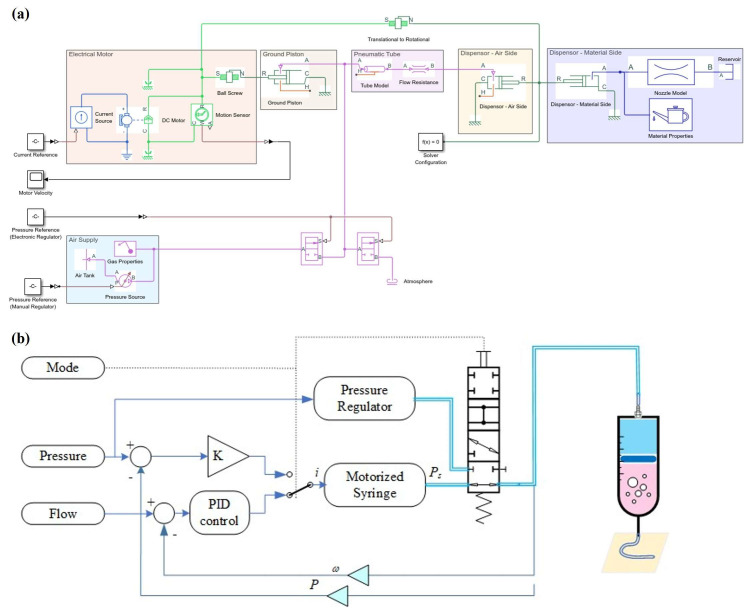
Digital twin co-simulation framework: (**a**) Simscape model and (**b**) control diagram.

In this section, a Simscape-based digital twin model is presented. The model integrates electromechanical, pneumatic, rheological, sensing, and control subsystems into a unified co-simulation framework, enabling rapid prototyping and closed-loop validation. This high-fidelity model, developed in MATLAB’s Simscape (2024a), supports real-time and energy-consistent simulations using variable-step solvers and parallel computing. Physical components such as the DC motor, ball screw, air reservoir, and flow elements are represented using bond graph abstractions (Figure 5a) and modular block diagrams (Figure 5b), capturing bidirectional energy flow and preserving their ordinary differential equation (ODE) formulations. Simulink integration also supports the implementation of custom control and sensing algorithms, with a tri-mode signal-flow control law closing the loop between simulation and hardware.

The model comprises five interlinked modules: (1) an electromechanical actuator module, which captures the dynamics of the DC motor and ball screw, (2) a pneumatic module, which models the air supply and transmission using Sutherland’s correction [28], (3) a material barrel module, simulating material flow via a lumped reservoir and nonlinear rheology, (4) a sensor interface, responsible for data acquisition and real-time streaming of $P_{S}$, $x_{C}$, $x_{S}$, and $x_{M}$, and (5) a control core module, which implements PID loops for reference tracking of $P_{S}$, $x_{C}$, and $\tau_{R}$. This framework enables all experiments presented in this study to be virtually reproduced without hardware changes.

### 2.6. Unified Dual-Mode Control

The unified control architecture integrates three control strategies (proportional, PID, and open loop) applied to pneumatic and mechanical elements to satisfy both transient and volumetric requirements. The proportional controller modulates an electropneumatic pressure regulator, ensuring that $P_{S}$ closely follows its setpoint with minimal lags. Concurrently, a PID loop governs DC motor velocity ${\dot{\theta }}_{R}$ in the motorized syringe, ensuring precise matching between ${\dot{x}}_{S}$ and the desired ${\dot{x}}_{M}$, which is derived from nozzle diameter and G-code commands. For system identification and simplified operation, an open-loop mode permits direct setting of both $P_{S}$ and ${\dot{x}}_{M}$ without relying on feedback. A supervisory scheduler manages these control strategies into four distinct operating modes: (1) piston-driven $P_{S}$ control, wherein a proportional–integral (PI) controller enforces $P_{S}$ by manipulating ${\dot{x}}_{C}$, (2) open-loop ${\dot{x}}_{M}$ control, in which a PID-driven controller manages ${\dot{x}}_{S}$ to deliver fixed values of ${\dot{x}}_{M}$ without feedback, (3) regulator-actuated $P_{S}$ control, where P-controlled valves maintain $P_{S}$ while ${\dot{x}}_{S}$ operates in an open loop, and (4) a synergistic pressure-flow mode that continuously blends together regulator and piston commands via weighted factors. This mode optimizes both transient damping and steady-state accuracy without requiring hardware changes.

## 3. Experimental Methodology

### 3.1. Materials and Test Metrics

To assess the performance of the system under varying material conditions, six experiments were conducted using materials characterized by distinct rheological properties. A standard acrylic paint (COLORPACK s.r.l., Solaro, Milano, Italy), known for its uniform rheological properties, was used as a homogeneous material to benchmark the system performance. To investigate the effects of material heterogeneity, a blend of two acrylic paints with different concentrations was employed. This heterogeneous mixture is composed primarily of pure acrylic paint into which discrete blobs of a second paint with a 90% w/w concentration are embedded, simulating nonuniform material rheology. Recognizing the critical influence of time-dependent rheology in extrusion, a two-component curing silicone resin was selected to observe how its viscoelastic properties change over time during curing. This material exhibits both elastic and viscous characteristics, enabling the assessment of system responses under viscoelastic transitions. Furthermore, to validate the system’s capability in forming functional geometries, a scaffold structure was fabricated using a self-curing acrylic sealant (3M, Saint Paul, MN, USA), which similarly undergoes time-dependent rheological changes. System versatility is further demonstrated through the extrusion of polypropylene, a thermoplastic polymer, under a semi-molten state at high temperatures. This material necessitates integration with a thermally controlled printhead, while the core actuation mechanism remains unchanged. Across all experimental conditions, system performance was consistently evaluated based on four quantitative metrics of printing linewidth w: coefficient of variation, starting overshoot, settling time, and directional asymmetry.

### 3.2. Instrumentation and Measurement Techniques

To evaluate extrusion performance, it is essential to quantify the deposited material and synchronize the measurements of w with the logged signals from the extrusion system. In this study, a Cartesian robot (Snapmaker 1.0, Snapmaker Inc., Shenzhen, China). is employed to establish a synchronization framework. This framework leverages both spatial positioning and the temporal sequencing of programmed deposition patterns for data alignment and harmonization. The extruded material was captured using an imaging technique based on a telecentric vision system, incorporating a Basler acA5472 camera (Ahrensburg, Germany) and a VS-5MPT0.095X208 telecentric lens (Vital Vision Technology Pte Ltd, Singapore). The captured images of the printed results were calibrated and registered to the robot’s coordinate system for spatial matching. Image registration consisted of two stages. First, the images were mapped to the physical world using a checkerboard calibration technique [29]. Second, the images were registered to the robot’s coordinate system using a template matching method. The template for image registration was generated from the robotic motion commands used for printing, ensuring accurate alignments between the printing patterns and programmed paths. Thus, the resulting lines were synchronized with the designated patterns in both space and time.

The volumetric flow rate of the extruded materials was estimated through w values. Extraction of w was performed by identifying the upper and lower edges of each printed line. By cropping individual lines, the resulting images were segmented. In the case of opaque materials, such as acrylic paints, paint mixtures, and silicone resins, color thresholding was employed for edge detection. For transparent materials, such as polypropylene, a dark-field imaging setup with 45-degree illumination was used to enhance edge visibility.

The extruder’s internal states were logged using an embedded microcontroller. The extruder was equipped with a quadrature encoder (GB37-EN, Yfrobot, SHENZHEN, China) and pressure sensor (ISE40-T1-22, SMC Corporation, Tokyo, Japan) The encoder was mounted on the motor rotor, allowing $\theta_{R}$ to be accurately measured. The measurement was used to infer $x_{C}$. With a sensing resolution of 8192 PPR and a 2 mm lead screw (NSK Ltd., Tokyo, Japan), this setup can attain an ideal resolution of 100 nm. A pressure sensor was installed on the pneumatic connection, continuously monitoring $P_{S}$. Pressure readings were digitized by an analog-to-digital converter on the microcontroller, achieving a resolution of 1 kPa. The microcontroller sampled both encoder and pressure data at 100 Hz. Using synchronization signals from the Cartesian robot, the recorded data was temporally aligned with the material deposition results.

### 3.3. Statistical Analysis Procedures

Statistical analysis was performed to quantify the stability, asymmetry, and repeatability of material extrusion across different actuation strategies and material rheology. For all experiments, the primary metric used to evaluate the stability of ${\dot{x}}_{M}$ was the coefficient of variation (CV) in w values, computed as the ratio of the standard deviation to the mean over each individual segment. Furthermore, CV was reported separately for forward and reverse passes to identify directional asymmetry. The characteristics of the transient response (overshoot and settling time of w) were also extracted by analyzing a series of w results at 1 kHz. Overshoot was defined as the maximum excursion above the steady-state mean within the first 500 ms of extrusion. Settling time was defined as the time interval to reach the steady state of w with a 5% tolerance. For materials with complex or time-dependent rheology, dynamic traces of the motor current i and $P_{S}$ were normalized and time-aligned to assess compensatory behavior and control-loop latency. Directional asymmetry was evaluated as the absolute percentage difference between w values from forward and reverse strokes. In scaffold printing, dimensional drift was assessed using layer-resolved pore measurements and expressed as the percentage deviation from the designed geometry.

All variability metrics were computed over a minimum dataset of ten independent printing strokes or twenty layers. The monotonicity and linearity of the actuation-to-result relationship (e.g. i to w) were determined through pairwise increments and visually inspected for curvature or saturation. These procedures enabled consistent benchmarking of each control strategy across variations in material rheology and solidification mechanisms while facilitating fair inter-experiment comparisons.

## 4. Experimental Results

In the experimental results, extruder performance was evaluated across six tests of ascending rheological complexity. The extrusion of an acrylic paint served to characterize the transient response and steady-state fidelity of w for homogeneous materials. Moreover, the extrusion of blended acrylic paint posed a challenge to the system due to its heterogeneous composition and varying rheological properties. Subsequent experiments involved extrusion of a two-component silicone resin, a material with time-dependent rheology under both fixed and adaptive control strategies on $P_{S}$. In scaffold printing, hybrid ${\dot{x}}_{C}$–$P_{S}$ control was applied to fabricate a multilayer structure using a self-curing material. Finally, temperature-controlled extrusion of polypropylene was conducted to determine the system’s versatility across distinct material domains.

### 4.1. Baseline Extrusion with Acrylic Paint

The primary objective of this experiment was to establish a quantitative benchmark for ${\dot{x}}_{M}$ stability and w fidelity prior to progressing to materials with more complex rheological behavior. A water-based acrylic paint was deposited using a 30 mL plastic syringe equipped with a 1.55 mm tapered nozzle (Figure 4c) Three actuation strategies were utilized: constant $P_{S}$, constant ${\dot{x}}_{C}$, and closed-loop i control. Material deposition included ten 60 mm lines, with i, $l_{C}$, and $P_{S}\;$ recorded in the log. Visual data of w was sampled at 1 kHz, thus enabling a full characterization of both transient and steady states.

**Figure 6:**
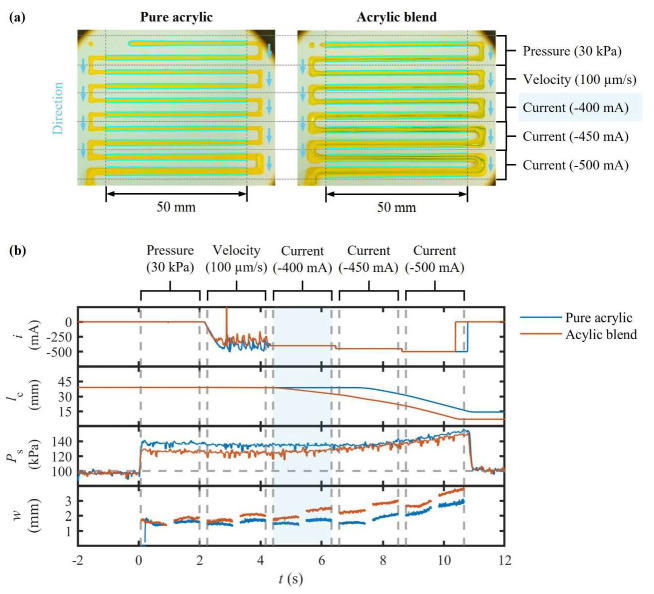
Results of line printing using a pure acrylic paint and an acrylic paint mixture: (**a**) top-view images of printed lines and (**b**) temporal response of motor current i, inner axial displacement of the pneumatic cylinder $l_{C}$, extrusion pressure $P_{S}$, and printed linewidth w.

The results reveal a clear performance hierarchy among the three actuation strategies (Figure 6). In pressure mode, air compressibility generated an overshoot of 35% and a high CV of 45% in the forward stroke, whereas the CV was reduced to 2.4% in the reverse stroke. The directional asymmetry was found to be 26%, highlighting the intrinsic hysteresis under constant $P_{S}$. Imposing a fixed ${\dot{x}}_{C}$ value reduced stochastic scatter (CV ≈ 3%), but retained a directional asymmetry of 14%, attributed to backlash in the ball-screw mechanism. In contrast, closed-loop i control decreased both overshoot (≤15%) and scatter at a setpoint of −400 mA. The mean value of w stabilized at 1.51 mm with a CV of 2.2% and settling time of 210 ms. Increasing the setpoint value of i to −450 mA and −500 mA produced systematically thicker w up to 2.77 mm while still maintaining CV values in the range of 2% to 6%. These findings demonstrate that i functioned as an effective throttle for ${\dot{x}}_{M}$ and w during extrusion. The slight nonlinearities observed beyond i of −450 mA suggest that the material exhibited shear thinning behavior once a critical stress threshold was surpassed. 

The dynamic analysis underscored the advantages of i regulation. Although all three regimes eventually converged to similar steady-state values of $P_{S}$, suggesting that the equilibrium of ${\dot{x}}_{M}$ was governed by the material’s inherent resistance to flow, their transient responses differed markedly. Constant $P_{S}\;$ dispensing released stored elastic energy in the pneumatic connection, leading to the slowest and most erratic convergence to steady-state conditions. The constant ${\dot{x}}_{C}$ mode avoided pneumatic lag but was still limited by mechanical compliance requiring time to overcome the sign-reversal dead zone. Closed-loop i control applied electrical force directly, allowing the target values of $P_{S}\;$ to be reached in one cycle, thus resulting in the fastest and most stable response.

These findings show that closed-loop i control combines rapid transient recovery with predictable, operator-tunable w scaling. This control strategy also reduces steady-state variability by an order of magnitude relative to conventional dispensing. This experiment therefore establishes practical reference levels for subsequent tests (CV ≈ 2% and a settling time < 250 ms). In addition, the results confirm that the proposed extruder, operated under closed-loop i control, outperforms conventional techniques based on invariant $P_{S}\;$ or ${\dot{x}}_{C}$, even when processing rheologically stable materials.

### 4.2. Heterogeneous Mixture Extrusion Under Rheology-Robust Control

The aim of this experiment was to ascertain whether this novel framework could maintain the stability of ${\dot{x}}_{M}$ achieved in the previous experiment. To this end, the material’s viscoelasticity was deliberately increased fourfold. Identical acrylic paint was mixed with droplets of another acrylic paint with a different concentration (90% *w*/*w*), thereby producing a heterogeneous material. The experiment followed the same protocol as the homogeneous paint extrusion. Hence, the results of this experiment are directly compared to the corresponding outcomes of the preceding experiment.

As shown in Figure 6, the stability of ${\dot{x}}_{M}$ remained remarkably resilient. Closed-loop i control constrained the CV to around 3% to 4%, marginally higher than the values observed with the homogeneous paint. In contrast, constant $P_{S}\;$ exhibited a forward CV of 5.6%, whereas constant ${\dot{x}}_{C}$ maintained a CV close to 3%. Likewise, the transient response confirmed the advantages of i regulation. The overshoot remained below 10% of the steady-state value, with a settling time of 250 s. This slightly longer settling time reflects the increased yield stress of the material, while the shrinkage of overshoot indicates that direct $\tau_{R}$ control is more effective in compensating for the material’s back pressure. Directional asymmetry emerged as the principal factor contributing to divergence. Under high load conditions, asymmetrical values rose to roughly 25%, compared to the range of 14% to 22% observed with pure acrylic paint. Moreover, the extrusion of reverse strokes reproduced the same magnitude of asymmetry. This observation confirms that this effect is attributable to mechanical backlash rather than material rheology. Notably, the i–w transfer function remained monotonic, with each 50 mA increase in i raising the mean w value by about 18%. The slight curvature below an i value of −500 mA demonstrates the onset of shear localization, occurring when local stress exceeds the break-up threshold of the pigment network. Meanwhile, control authority is preserved.

These findings highlight that the proposed extruder sustains w variability below 6%, achieves a settling time of under 300 ms, and maintains a predictable relationship between i and w even with a fourfold increase in viscosity. The only substantive degradation (directional asymmetry) issues arose from recoverable transmission backlash rather than from the material. Consequently, this experiment validates the rheology-robust control of the proposed architecture, effectively extending its operational range to more challenging materials without necessitating controller retuning.

### 4.3. Constant-Pressure Extrusion of a Curing Two-Component Silicone Resin 

This experiment was designed to illustrate the limitations of constant $P_{S}$ in extruding curing materials whose viscoelasticity develops over time. A commercial two-component silicone resin (pot life ≈ 45 min at 25 °C) was mixed 30 min prior to extrusion every 5 min under a constant $P_{S}$ value of 150 kPa. Continuous data logging was applied for i, $l_{C}$, and $P_{S}$. Subsequently, top-view images of the printed results were captured in chronological order to extract w values.

**Figure 7:**
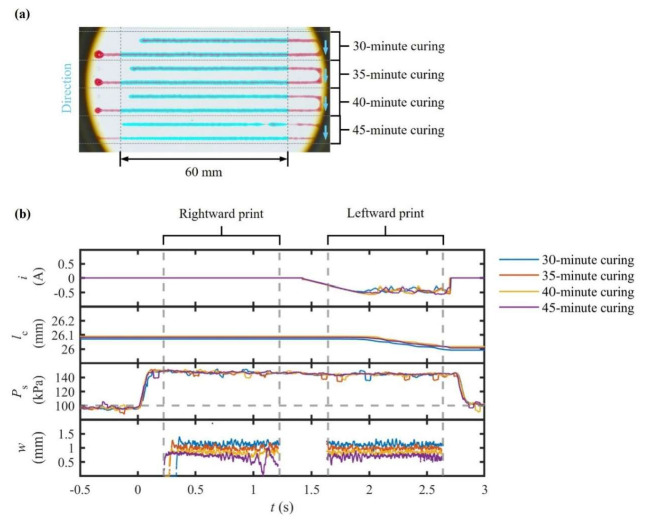
Line printing results for a curing two-component silicon resin under constant pressure: (**a**) top-view image of a printed line and (**b**) temporal responses of motor current i, inner axial displacement of the pneumatic cylinder $l_{C}$, extrusion pressure $P_{S}$, and printed linewidth w.

In Figure 7, the results reveal a monotonic decline in ${\dot{x}}_{M}$. During the initial test, the mean value of w was measured as 1.06 ± 0.07 mm (CV = 6.6%). By the final test, conducted 45 min after mixing, this value had fallen to 560 ± 160 µm (CV = 28%). Throughout the experiment, the measured values of $P_{S}$ remained within ±3.4% of the target setpoint, while no compensatory rise was observed in i. These results confirm that the hardware continued to supply the programmed $P_{S}$ value even as the material itself exhibited increasing resistance to flow. Moreover, $l_{C}$ decayed gradually, indicating that its decline delivered progressively less material. 

The interpretation is thus unequivocal. Material extrusion with constant $P_{S}$ fails to accommodate the developing viscoelastic characteristics of curing materials. Since ${\dot{x}}_{M}$ increases inversely to viscosity under constant $P_{S}$, an increase in viscosity produced a near-half value of w coupled with a disproportionate rise in variability, despite both $P_{S}$ and i appearing nominal. A comparison between the preceding experiments is instructive: i regulation maintained a CV below 6% for both benign and highly viscous materials, excluding curing inks. Constant $P_{S}$ left the time-dependent effects uncompensated for, proving inadequate for materials with evolving rheology.

Two key implications are also revealed. Firstly, to preserve dimensional fidelity, reactive materials with a short curing time require either a pre-programmed $P_{S}$ trajectory or the integration of volumetric and force feedback control. Secondly, the data establish a practical upper limit of about 40 min for continuous printing, after which a constant $P_{S}$ strategy can no longer maintain uniform deposition without modification. These insights play a critical role in guiding process planning for structural adhesion, electronic potting, and composite repair, where in situ curing is unavoidable and precision is paramount.

### 4.4. Adaptive Pressure Extrusion of a Curing Two-Component Silicone Resin

The objective of this experiment was to establish whether feedback control in $P_{S}$ could counteract rapid viscosity increases during the curing of materials. The same two-component silicone resin was utilized with identical printing timings. However, in this case, $P_{S}$ was allowed to compensate for deviations in w. Throughout the experiment, i, $l_{C}$, and $P_{S}$ were continuously monitored to assess the effectiveness of the control system and to track the material’s behavior during in situ curing.

**Figure 8:**
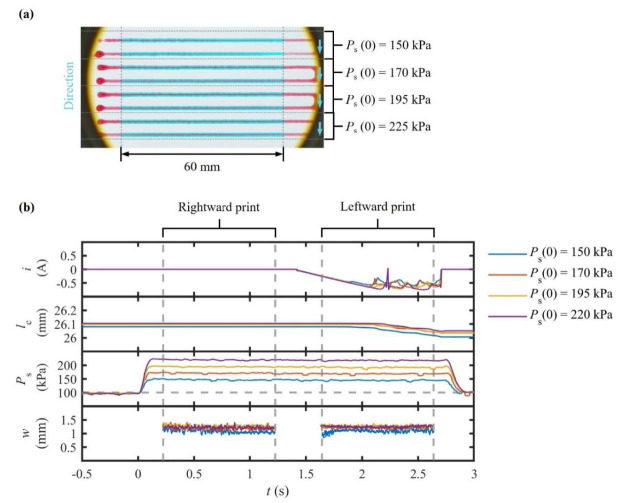
Line printing results for a cured two-component silicon resin under adaptive pressure control: (**a**) top-view image of a printed line and (**b**) temporal response of motor current i, inner axial displacement of the pneumatic cylinder $l_{C}$, extrusion pressure $P_{S}$, and printed linewidth w.

The results demonstrate a pronounced improvement in dimensional fidelity (Figure 8). Over a 15 min period, $P_{S}$ increased linearly from 150 to 220 kPa (≈47%), while i increased by only 15%. This discrepancy indicates that pneumatic effects offer greater efficacy than mechanical responses in monitoring load stability during curing. At steady state, w stabilized at 1.18 ± 0.04 mm, remaining within ±3.5% of its moving average. Notably, the CV never exceeded 4%. In contrast, a 54% contraction of w was observed, accompanied by a fourfold increase in CV. As for the transient response, overshoot stayed below 8% with a settling time of the order of 250 ms.

These findings imply that moderate increments in $P_{S}$ effectively counteract the time-dependent rheological evolution of curing resins. Meanwhile, the impact of i remains comparatively limited. The direct correlation between instantaneous $P_{S}$ and w confirms that the adaptive schedule governs ${\dot{x}}_{M}$ effectively, restoring consistent deposition without introducing directional asymmetry as observed under constant i.

The implications of this study go beyond its immediate scope. The results show that reactive materials with a short curing time can be processed via existing pneumatic dispensers by making software modifications, avoiding the need for costly hardware upgrades. The proposed architecture provides an alternative way of operating when volumetric or force feedback is unavailable. These findings underscore the versatility and scalability of adaptive $P_{S}$ control in processing time-dependent and high-modulus materials, robustly closing the gap between simple pneumatic dispensing and torque-based extrusion.

### 4.5. Scaffold Printing with a Self-Curing Sealant Under Combined Velocity–Pressure Control

This experiment was conducted to evaluate whether the proposed architecture could sustain strand uniformity and pore fidelity during the fabrication of a multilayer structure for a self-curing sealant. For this purpose, the extrusion system was operated in a hybrid mode, utilizing a fixed ${\dot{x}}_{C}$ value of 100 µm/s and an initial $P_{S}$ value of 115 kPa to ensure immediate material flow. This configuration decouples steady-state volumetric delivery, regulated by the screw mechanism, from short-term rheological fluctuations buffered in the pneumatic connection. Throughout the process, four performance indicators were monitored synchronously: (1) $P_{S}$ decays, representing increased material flow resistance; (2) compensatory i increments, quantifying the $\tau_{R}$ margin required to maintain ${\dot{x}}_{S}$; (3) statistical metrics of w, including CV and bidirectional asymmetry; and (4) layer-resolved pore geometry, obtained via visual profilometry, assessing cumulative dimensional drift over the build height.

### 4.6. Temperature-Controlled Extrusion of Polypropylene

**Figure 9:**
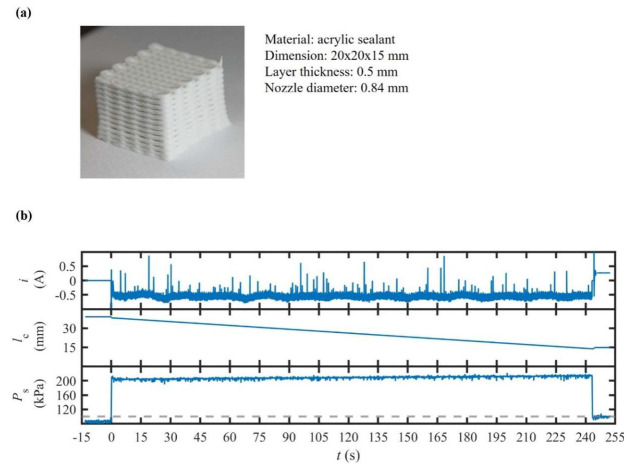
The results of scaffold printing using a self-curing acrylic sealant: (**a**) isometric image of the fabricated structure and (**b**) temporal responses of the motor current i, the inner axial displacement of the pneumatic cylinder $l_{C}$, and the extrusion pressure $P_{S}$.

In Figure 9, the results show that $P_{S}$ declined gradually by 9 kPa over the course of twenty layers, while i remained at ±8% of its nominal value. This behavior indicates that ${\dot{x}}_{C}$ and the preload value of $P_{S}$ were well-matched to the material’s solidification kinetics. It can be seen that the measured value of w stayed within a ±5% range (CV ≤ 4%) and directional asymmetry was kept under 6%, which is significantly lower than comparable results for the extrusion of acrylic paint mixtures. For the top layer, the pore size deviated by 4% from its designated value, with no evidence of structural collapse or pore closure detected.

Such observations confirm that the proposed extrusion system can maintain dimensional accuracy and repeatability throughout the construction of multilayer structures with curing materials when a combined velocity–pressure mode is applied. Such findings extend the applicability of extrusion from benign and silicone-based inks to self-curing materials, highlighting its potential for construction-scale sealing, repair applications, and the fabrication of soft-matter scaffolds, where in situ solidification is unavoidable.

In this experiment, the system’s capability to extrude a thermoplastic polymer was evaluated, using a custom heat-jacketed syringe dispenser (Figure 4d). A 10 mL metal syringe was fitted with a cartridge heater and thermistor; to maintain temperature, its outlet was attached to a heat block. To ensure thermal uniformity, an extra heater and thermistor were installed on this heat block. A 400 µm brass nozzle, (Creality, Shenzhen, China) was connected to the heat block to adjust the outlet size. 

**Figure 10:**
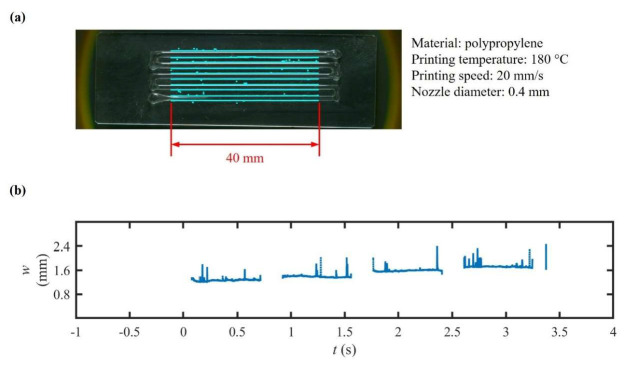
Line printing for polypropylene: (**a**) top-view image of printed line and (**b**) temporal response of printed linewidth w.

Polypropylene was selected as the printing material. Regulated $P_{S}$ drove $l_{S}$ under open-loop volumetric control. The printing temperature kept held at 180 ± 2 °C, and the printing speed was maintained at 20 mm/s. Throughout the printing process, nozzle temperature, $P_{S}$, $l_{C}$, and i were logged while telecentric imaging metrology was utilized to capture the values of w. The resulting values of w averaged 1.57 ± 0.21 mm (CV = 13.4%), with overshoots below 15% and a settling time of under 300 ms (Figure 10).

The observed increase in the CV, compared to low-temperature extrusion, reflects the presence of thermal gradients within the fused material and the lack of closed-loop compensation in $P_{S}$ during the process. Accordingly, future studies should integrate active thermal feedback and pressure control for high-fidelity thermoplastic DIW.

## 5. Conclusions

This paper presents a dual-mode electropneumatic extrusion architecture capable of real-time adaptation to diverse material rheology through hybrid pressure–displacement control. By integrating a motorized pneumatic cylinder with an electropneumatic pressure regulator, this system mitigates viscoelastic lag, pneumatic compliance, and mechanical backlash, which are common limitations in DIW. A Simscape-based digital twin, enhanced with extended Kalman filtering, enables continuous synchronization with the physical system for online parameter identification and state estimation without additional hardware. Experimental validation across five material types (homogeneous, heterogeneous, time-dependent rheology, self-curing, and thermoplastic) demonstrates consistent linewidth control (CV ≤ 4%) and a settling time of below 250 ms, significantly lower than the performance of conventional pressure or feed rate control alone in comparable rheological conditions (CV > 10%). Adaptive pressure ramping improved print fidelity over material curing, while hybrid control maintained dimensional accuracy in multilayer fabrication. The analysis of dynamic responses showed that motor current control and adaptive pressure address distinct transient response characteristics (overshoot, hysteresis, and compliance delay), with remaining directional asymmetry linked to ball-screw backlash. These findings offer practical guidance for control-mode selection (current control, pressure control, and hybrid schemes), based on rheological behavior and process requirements. Furthermore, the digital twin provides a scalable and platform-agnostic tool for prototyping and strategy optimization. This work establishes a foundation for the future integration of high-bandwidth sensing, model-predictive control, and scalable multi-nozzle DIW systems, advancing high-fidelity and rheology-adaptive additive manufacturing.

## Figures and Tables

**Table 1:** Simulation parameters and boundary conditions for the extrusion of a custom liquid material.

Parameters	Values
Simulation time	15 s
Sampling time	100 ms
Mesh size	50–980 µm
${\dot{x}}_{C}$	5 mm/s
$D_{C}$	16 mm
$l_{C}$	200 mm
$D_{S}$	22.5 mm
$l_{S}$	40 mm
$V_{D}$	1.256 L
Material density	1.2 Gg/m^3^
Material viscosity	20 Pa·s
Material thermal conductivity	200 mW/(m·K)
Material heat capacity	2 kJ/(kg·K)
Wall thermal conductivity	Perfect insulation
Wall slip condition	Slip
Initial temperature	300 K
Initial pressure	$P_{atm}$ (101.325 kPa)

## Data Availability

The data supporting the findings of this study is available on request.

## Abbreviations

**Symbols**	**Descriptions**
$A_{C},A_{S}$	inner cross-sectional area of cylinder and syringe
$c_{M}$,$c_{O}$	viscous coefficient of printing material damping and flow resistance
$D_{C},D_{S}$	inner diameter of cylinder and syringe
$D_{W}$	screw nominal diameter
$F_{M},F_{V}$	printing material flow resistance and internal viscoelasticity force
$F_{C},F_{S}$	pneumatic force on pneumatic cylinder and syringe piston
$F_{W}$	screw load
$f_{T},f_{O}$	static and viscous components of flow resistance
G	printing material shear modulus
i	motor current
$J_{R},J_{W}$	moment of inertia of motor and screw
$k_{M}$	printing material spring coefficient
$l_{C},l_{S}$	inner axial displacement of cylinder and syringe
$m_{C},m_{S}{,m}_{D}$	air mass in cylinder, syringe, and dead volume
$m_{P},m_{M}{,m}_{W}$	mass of syringe piston, printing material, and screw carriage
$P_{atm}$	absolute atmospheric pressure
$P_{C},P_{S}{,P}_{D}$	absolute air pressure in cylinder, syringe, and dead volume
t	time
$T_{C},T_{S}{,T}_{D}$	air temperature in cylinder, syringe, and dead volume
$V_{C},V_{S}{,V}_{D}$	air volume in cylinder, syringe, and dead volume
w	linewidth of printed material
$x_{C},x_{S}$	linear displacement of cylinder and syringe piston
$x_{M},x_{W}$	linear displacement of printing material entering nozzle and screw
α	screw lead angle
β	screw friction angle
γ	shear strain of printing material
η	printing material viscosity
$\theta_{R},\theta_{W}$	angular displacement of motor and screw
μ	screw friction coefficient
$\rho_{C}{,\rho }_{S}{,\rho }_{D}$	air density in cylinder, syringe, and dead volume
$\rho_{M}$	printing material density
σ	shear stress of printing material
$\tau_{R},\tau_{W}$	torque of motor and screw
